# Supplementary material for: Investigating the role of the relaxin-3/RXFP3 system in neuropsychiatric disorders and metabolic phenotypes: A candidate gene approach
Source: PLoS One. 2023 Nov 15;18(11):e0294045. doi: 10.1371/journal.pone.0294045 (PMC10651050; doi:10.1371/journal.pone.0294045)
Supplement: S1 Table — (DOCX) [file pone.0294045.s001.docx]

**Supplementary Table 1**: Full description and field codes used to derive phenotype caseness definitions for depression and atypical depression.

| **Depression Phenotypes** | **Caseness description** | **Relevant data field codes** |
| --- | --- | --- |
| Broad depression | Answered “yes” to the question “Have you ever seen a GP for nerves, anxiety, tension or depression”  **OR**  Answered “yes” to the question “Have you ever seen a GP/psychiatrist for nerves, anxiety, tension or depression”  **OR**  Have a primary or secondary diagnosis of one of the following ICD-10 codes for mood disorders in hospital episode data from UK bodies:   - F32: depressive episode - F33: recurrent depressive disorder - F34: persistent mood disorders - F38: other mood disorders - F39: unspecified mood disorder | 2090, 2100  41202, 41204 |
| ICD10-coded depression | Have a primary or secondary diagnosis of one of the following ICD-10 codes for mood disorders in hospital episode data from UK bodies:   - F32: depressive episode - F33: recurrent depressive disorder - F34: persistent mood disorders - F38: other mood disorders - F39: unspecified mood disorder | 41202, 41204 |
| Lifetime depression | Indication of major depression status from derived data field generated by DJ Smith et al.^1^, based on touchscreen responses for help-seeking behaviour and extended presence of low mood or anhedonia | 20126 |
| CIDI depression | Met all the following criteria:   1. Answered “yes” to having experienced anhedonia or low mood for two weeks or more in a row 2. Answered “yes” to having 4 or more of the following 8 symptoms, based on the CIDI assessment:    1. Anhedonia    2. Low mood    3. Trouble with concentration    4. Ideation of death    5. More tired or low on energy than usual    6. Feelings of worthlessness    7. Trouble falling asleep, sleeping too much, or waking too early    8. Gaining or losing weight 3. Answered “almost every day” or “every day” [coding 3] to a question about the frequency of depressed days during their worst episode of depression. 4. Answered either “about half of the day”, “most of the day”, or “all day long” to the question “How much of the day did these feelings usually last?" during their worst episode of depression. 5. Answered “somewhat” [coding 2] or “a lot” [coding 3] to the following prompt: "Think about your roles at the time of this episode, including study/employment, childcare and housework, leisure pursuits. How much did these problems interfere with your life or activities?", during their worst episode of depression | 20446, 20441  20435, 20437, 20449, 20450, 20533, 20534, 20535, 20536  20439, 20436, 20440 |
| PHQ-9 definition depression | Questions from the PHQ-9 screening tool were administered in an online mental health follow up questionnaire.  Cases met all the following criteria:   1. Answered “more than half the days” or “nearly every day” when asked how often they had been bothered by either anhedonia or low mood. 2. Answered “more than half the days” or “nearly every day” for 5 or more of the following symptoms, when asked how often they had been bothered by said symptom:    1. Lack of interest or pleasure in doing things    2. Depression    3. Trouble concentrating on things    4. Thoughts of suicide or self-harm    5. Feelings of tiredness or low energy    6. Feelings of inadequacy    7. Trouble falling asleep, sleeping too much, or waking too early    8. Poor appetite or overeating    9. Changes in speed/amount of moving or speaking | 20514, 20510  20507, 20508, 20517, 20518, 20519, 20511, 20513 |
| PHQ-9 cutoff depression | Questions from the PHQ-9 screening tool were administered in an online mental health follow up questionnaire.  The presence of each of the following symptoms was rated on a scale of 1-4, analogous to the PHQ-9 ratings of 0-3. 1 corresponds to not at all, 2 corresponds to several days, 3 corresponds to more than half the days, and 4 corresponds to nearly every day:   1. Recent feelings of depression 2. Recent lack of interest or pleasure in doing things 3. Recent feelings of tiredness or low energy 4. Recent trouble concentrating on things 5. Recent thoughts of suicide or self-harm 6. Recent poor appetite or overeating 7. Trouble falling or staying asleep, or sleeping too much 8. Recent feelings of inadequacy 9. Recent changes in speed/amount of moving or speaking   Cases had a score of ≥19, corresponding to the PHQ-9 cutoff score of ≥10. | 20514, 20510  20507, 20508, 20517, 20518, 20519, 20511, 20513 |
| CIDI atypical depression | Met case status on the CIDI depression phenotype definition  **AND**  Answered “yes” to sleeping too much during worst period of depression or answered “gained weight” when asked about weight change during worst episode of depression. | 20446, 20441, 20435, 20437, 20449, 20450, 20533, 20534, 20535, 20536, 20439, 20436, 20440  20536, 20534 |
| PHQ-9 definition atypical depression | Met case status on the PHQ-9 definition phenotype of depression  **AND**  Answered “yes” to sleeping too much during worst period of depression or answered “gained weight” when asked about weight change during worst episode of depression. | 20514, 20510, 20507, 20508, 20517, 20518, 20519, 20511, 20513  20536, 20534 |
| PHQ-9 cutoff atypical depression | Met case status on the PHQ-9 cutoff phenotype of depression  **AND**  Answered “yes” to sleeping too much during worst period of depression or answered “gained weight” when asked about weight change during worst episode of depression. | 20514, 20510, 20507, 20508, 20517, 20518, 20519, 20511, 20513  20536, 20534 |
