# Supplementary material for: Investigating the role of the relaxin-3/RXFP3 system in neuropsychiatric disorders and metabolic phenotypes: A candidate gene approach
Source: PLoS One. 2023 Nov 15;18(11):e0294045. doi: 10.1371/journal.pone.0294045 (PMC10651050; doi:10.1371/journal.pone.0294045)
Supplement: S2 Table — (DOCX) [file pone.0294045.s002.docx]

**Supplementary Table 2**: Full description and field codes used to derive phenotype caseness definitions for anxiety.

| **Anxiety Phenotypes** | **Description** | **Relevant field codes** |
| --- | --- | --- |
| ICD10-coded anxiety | Have a primary or secondary diagnosis of one of the seven following ICD-10 codes for anxiety disorders in hospital episode data from UK bodies:   - F40: phobic anxiety disorders - F41: other anxiety disorder - F42: obsessive-compulsive disorder - F43: reaction to severe stress, and adjustment disorders - F44: dissociative disorders - F45: somatoform disorders - F48: other neurotic disorders | 41202, 41204 |
| Lifetime disorder anxiety | Met all the following criteria:   1. Answered yes to either of the following questions: "People differ a lot in how much they worry about things. Did you ever have a time when you worried a lot more than most people would in your situation?" **or** "Please think of the period in your life when you have felt worried, tense, anxious, or more worried than most people would in your situation. This could be in the past, or it could be continuing now. During that period, was your worry stronger than in other people?" 2. Answered yes to the question "Have you ever had a period lasting one month or longer when most of the time you felt worried, tense, or anxious?" 3. Had this frequent and persistent worrying for 6 months or longer 4. Answered yes to the question “Did you worry most days?" during their worst period of anxiety 5. Answered yes to either of the following questions: " Did you ever have different worries on your mind at the same time?" **or** "Did you usually worry about one particular thing, such as your job security or the failing health of a loved one, or more than one thing?" during their worst period of anxiety 6. Answered yes to the question “Did you find it difficult to stop worrying?" **or** answered “often” to the question “How often was your worry so strong that you couldn't put it out of your mind no matter how hard you tried?" **or** answered “often” to the question “How often did you find it difficult to control your worry?" 7. Answered “a lot” to the question "Think about your roles at the time of this episode, including study / employment, childcare and housework, leisure pursuits. How much did these problems interfere with your life or activities?" 8. Suffered 3 or more somatic symptoms of anxiety during their worst period of anxiety (including feelings of restlessness; feeling easily tired; having trouble falling or staying asleep; feeling keyed up or on edge; increased irritability; experiencing tense, sore or aching muscles; having difficulty concentrating)   **OR**  Self-reported receiving a professional diagnosis of one or more of the following disorders:   - Social anxiety or social phobia - Any other phobia (eg disabling fear of heights or spiders) - Panic attacks - Obsessive compulsive disorder (OCD) - Anxiety, nerves or generalized anxiety disorder - agoraphobia | 20425, 20542, 20426, 20429, 20427, 20423, 20422, 20417, 20419  20421, 20420, 20538, 20540, 20543, 20541, 20539, 20537  20544 |
| GAD-7 cutoff anxiety | Questions from the GAD-7 screening tool were administered in an online mental health follow up questionnaire. The presence of each of the following symptoms was rated on a scale of 1-4, analogous to GAD-7 ratings of 1-3. 1 corresponds to not at all, 2 corresponds to several days, 3 corresponds to more than half the days, and 4 corresponds to nearly every day:   1. Recent easy annoyance or irritability 2. Recent feelings of foreboding 3. Recent feelings or nervousness or anxiety 4. Recent inability to stop or control worrying 5. Recent restlessness 6. Recent trouble relaxing 7. Recent worrying too much about different things   Cases had a score of ≥17, corresponding to the GAD-7 cutoff score of ≥10. | 20505, 20512, 20506, 20509, 20516, 20515, 20520 |
