# Supplementary material for: Investigating the role of the relaxin-3/RXFP3 system in neuropsychiatric disorders and metabolic phenotypes: A candidate gene approach
Source: PLoS One. 2023 Nov 15;18(11):e0294045. doi: 10.1371/journal.pone.0294045 (PMC10651050; doi:10.1371/journal.pone.0294045)
Supplement: S3 Table — (DOCX) [file pone.0294045.s003.docx]

**Supplementary Table 3**: Full description and field codes used to derive phenotype caseness definitions for metabolic syndrome and the sub-outcomes that comprise the disorder.

| **Metabolic Phenotypes** | **Description** | **Relevant field codes** |
| --- | --- | --- |
| High waist circumference | Waist circumference of ⩾102 cm for males or ⩾88 cm for females | 48 |
| Hypertension | Systolic blood pressure ⩾130 mmHg or diastolic blood pressure ⩾85 mmHg  **OR**  On one of the following antihypertensive drug treatments, based on participant self-reported medication data:   - Doxazosin - Clonidine - Moxonidine - Indoramin - Methyldopa - Prazosin - Minoxidil - Hydralazine - Bendroflumethiazide - Atenolol - Bisoprolol - Furosemide - Propranolol - Indapamide - Spironolactone - Metoprolol - Sotalol - Chlorthalidone \|Atenolol - Amiloride \|Furosemide - Nebivolol - Bumetanide - Timolol - Carvedilol - Bisoprolol \|Hydrochlorothiazide - Amiloride \|Hydrochlorothiazide - Amiloride - Celiprolol - Bendroflumethiazide \|Potassium - Atenolol \|Bendroflumethiazide - Eplerenone - Labetalol - Triamterene \|Hydrochlorothiazide - Bendroflumethiazide \|Propranolol - Hydrochlorothiazide - Chlorthalidone - Sotalol \|Hydrochlorothiazide - Carteolol - Betaxolol - Amiloride \|Cyclopenthiazide - Atenolol \|Nifedipine - Metoprolol \|Chlorthalidone - Acebutolol - Oxprenolol - Atenolol \|Amiloride \|Hydrochlorothiazide - Nadolol - Torasemide - Metolazone - Xipamide - Pindolol - Cyclopenthiazide - Ramipril - Amlodipine - Lisinopril - Perindopril - Candesartan - Losartan - Felodipine - Irbesartan - Enalapril - Valsartan - Lercanidipine - Nifedipine - Diltiazem - Verapamil - Telmisartan - Olmesartan - Lacidipine - Losartan \|Hydrochlorothiazide - Trandolapril - Eprosartan - Fosinopril - Captopril - Quinapril - Lisinopril \|Hydrochlorothiazide - Hydrochlorothiazide \|Irbesartan - Enalapril \|Hydrochlorothiazide - Perindopril \|Indapamide - Valsartan \|Hydrochlorothiazide - Diltiazem \|Hydrochlorothiazide - Nicardipine - Telmisartan \|Hydrochlorothiazide - Ramipril \|Felodipine - Imidapril - Cilazapril - Hydrochlorothiazide \|Captopril | 4079, 4080, 20003 |
| Hypertriglyceridaemia | Triglycerides level ⩾150 mg/dL  **OR**  On one of the following drug treatments for elevated triglycerides, based on participant self-reported medication data:   - Fenofibrate - Bezafibrate - Ciprofibrate - Gemfibrozil - Ezetimibe - Fish Oil - Omega-3-Acid Ethyl Esters - Simvastatin - Atorvastatin - Rosuvastatin - Pravastatin - Fluvastatin | 30870, 20003 |
| Low HDL cholesterol  (Dyslipidaemia) | HDL cholesterol levels < 40 mg/dL for men or < 50 mg/dL for women  **OR**  On one of the following drug treatments for reduced HDL cholesterol, based on participant self-reported medication data:   - Naftidrofuryl Oxalate - Niacin - Pentoxifylline - Simvastatin - Atorvastatin - Rosuvastatin - Pravastatin - Fluvastatin - Fenofibrate - Bezafibrate - Ciprofibrate - Gemfibrozil - Acipimox | 3076, 20003 |
| Hyperglycaemia | Fasting glucose ⩾100 mg/dL  **OR**  On one of the following drug treatments for elevated glucose, based on participant self-reported medication data:   - Metformin - Insulin - Gliclazide - Pioglitazone - Rosiglitazone - Glimepiride - Metformin \|Rosiglitazone - Glyburide - Glipizide - Repaglinide - Tolbutamide - Acarbose - Nateglinide | 30740, 20003 |
| Metabolic syndrome | Case status for at least three of the five metabolic outcomes defined above:   1. High waist circumference 2. Hypertension 3. Hypertriglyceridemia 4. Low HDL cholesterol 5. Hyperglycemia | All field codes listed in rows above |
