# Supplementary material for: Investigating the role of the relaxin-3/RXFP3 system in neuropsychiatric disorders and metabolic phenotypes: A candidate gene approach
Source: PLoS One. 2023 Nov 15;18(11):e0294045. doi: 10.1371/journal.pone.0294045 (PMC10651050; doi:10.1371/journal.pone.0294045)
Supplement: S4 Table — Regression models were adjusted for age, age2, sex, genotyping batch, testing centre, and the first six European ancestry principal components. Unadjusted p values and q-values (calculated by applying false discovery rate correction across phenotype definitions) are presented. (DOCX) [file pone.0294045.s004.docx]

**Supplementary Table 4:** Full associations between each candidate SNP and each of the 6 phenotypic definitions for depression. Regression models were adjusted for age, age^2^, sex, genotyping batch, testing centre, and the first six European ancestry principal components. Unadjusted p values and q-values (calculated by applying false discovery rate correction across phenotype definitions) are presented.

| **SNP** | **A1/A2** | **Broad** | | | **ICD10-coded** | | | **Lifetime** | | | **CIDI** | | | **PHQ-9 definition** | | | **PHQ-9 cutoff** | | |
| --- | --- | --- | --- | --- | --- | --- | --- | --- | --- | --- | --- | --- | --- | --- | --- | --- | --- | --- | --- |
|  |  | **B (Std. Error)** | ***P*** | **q-value** | **B (Std. Error)** | ***P*** | **q-value** | **B (Std. Error)** | ***P*** | **q-value** | **B (Std. Error)** | ***P*** | **q-value** | **B (Std. Error)** | ***P*** | **q-value** | **B (Std. Error)** | ***P*** | **q-value** |
| rs1982632 | A/G | 0.00131 (0.00639) | 0.837 | 0.954 | -0.00079 (0.0137) | 0.954 | 0.954 | 0.0017 (0.0143) | 0.905 | 0.954 | -0.0142 (0.0122) | 0.247 | 0.74 | -0.0185 (0.0257) | 0.473 | 0.946 | -0.0407 (0.0254) | 0.109 | 0.652 |
| rs78161395 | T/G | -0.0139 (0.00684) | 0.0428 | 0.257 | -0.003 (0.0147) | 0.838 | 0.919 | -0.0112 (0.0152) | 0.463 | 0.919 | -0.00133 (0.013) | 0.919 | 0.919 | 0.0105 (0.0272) | 0.698 | 0.919 | 0.0163 (0.0266) | 0.539 | 0.919 |
| rs74400983 | T/C | 0.0248 (0.0109) | 0.0232 | 0.139 | 0.0206 (0.0232) | 0.376 | 0.654 | 0.0369 (0.0242) | 0.127 | 0.381 | -0.0127 (0.0209) | 0.545 | 0.654 | -0.0323 (0.044) | 0.462 | 0.654 | -0.0136 (0.043) | 0.752 | 0.752 |
| rs6511905 | G/C | -0.00102 (0.00587) | 0.862 | 0.989 | 0.00367 (0.0126) | 0.77 | 0.989 | 0.000182 (0.0131) | 0.989 | 0.989 | -0.00582 (0.0112) | 0.604 | 0.989 | 0.00214 (0.0234) | 0.927 | 0.989 | 0.00387 (0.0229) | 0.866 | 0.989 |
| rs9292519 | A/G | -0.00883 (0.00508) | 0.0822 | 0.247 | -0.0105 (0.0109) | 0.335 | 0.67 | -0.00722 (0.0113) | 0.524 | 0.787 | -0.0182 (0.00973) | 0.0611 | 0.247 | -0.00631 (0.0203) | 0.756 | 0.865 | 0.00338 (0.0199) | 0.865 | 0.865 |
| rs171631 | A/C | 0.00469 (0.0106) | 0.658 | 0.789 | -0.0192 (0.0229) | 0.402 | 0.604 | 0.00343 (0.0236) | 0.885 | 0.885 | -0.0384 (0.0203) | 0.0582 | 0.349 | -0.0418 (0.0429) | 0.33 | 0.604 | -0.04 (0.0421) | 0.342 | 0.604 |
| rs42868 | G/C | 0.00317 (0.00684) | 0.643 | 0.749 | -0.00515 (0.0147) | 0.725 | 0.749 | -0.0163 (0.0153) | 0.286 | 0.749 | -0.0167 (0.0131) | 0.203 | 0.749 | 0.00869 (0.0272) | 0.749 | 0.749 | 0.0225 (0.0266) | 0.398 | 0.749 |
| rs7702361 | A/C | 0.00836 (0.00509) | 0.1 | 0.217 | 0.00462 (0.0109) | 0.672 | 0.806 | 0.0183 (0.0114) | 0.108 | 0.217 | 0.0191 (0.00974) | 0.0499 | 0.217 | 0.00065 (0.0203) | 0.974 | 0.974 | -0.0219 (0.02) | 0.274 | 0.41 |
| rs11264422 | T/A | 0.00591 (0.00528) | 0.263 | 0.621 | 0.0114 (0.0113) | 0.311 | 0.621 | 0.0155 (0.0118) | 0.187 | 0.621 | -0.00405 (0.0101) | 0.688 | 0.825 | -0.00386 (0.021) | 0.854 | 0.854 | -0.00854 (0.0207) | 0.679 | 0.825 |
| rs62351166 | A/C | 0.0135 (0.00656) | 0.0388 | 0.233 | 0.0148 (0.014) | 0.29 | 0.435 | 0.0156 (0.0147) | 0.289 | 0.435 | 0.000899 (0.0126) | 0.943 | 0.943 | -0.0299 (0.0267) | 0.262 | 0.435 | 0.00487 (0.0259) | 0.851 | 0.943 |
| rs7695640 | G/A | 0.00748 (0.00719) | 0.298 | 0.894 | 0.000788 (0.0154) | 0.959 | 0.959 | 0.00268 (0.0161) | 0.868 | 0.959 | 0.0193 (0.0138) | 0.16 | 0.894 | -0.0125 (0.029) | 0.665 | 0.959 | -0.0177 (0.0285) | 0.535 | 0.959 |
| rs11100192 | G/A | 0.0225 (0.0225) | 0.318 | 0.747 | 0.0352 (0.0475) | 0.458 | 0.747 | 0.0457 (0.05) | 0.361 | 0.747 | -0.0147 (0.0428) | 0.731 | 0.747 | 0.0285 (0.0885) | 0.747 | 0.747 | -0.032 (0.089) | 0.719 | 0.747 |
| rs72703633 | C/T | 0.0187 (0.0234) | 0.423 | 0.896 | 0.0259 (0.0497) | 0.603 | 0.896 | 0.0485 (0.0511) | 0.343 | 0.896 | 0.00877 (0.0443) | 0.843 | 0.896 | 0.012 (0.0916) | 0.896 | 0.896 | 0.0518 (0.0889) | 0.56 | 0.896 |
| rs11793069 | G/A | -0.0027 (0.00503) | 0.592 | 0.887 | -0.018 (0.0108) | 0.0946 | 0.568 | -0.00144 (0.0112) | 0.898 | 0.898 | 0.00399 (0.00959) | 0.677 | 0.887 | 0.00905 (0.02) | 0.651 | 0.887 | -0.00654 (0.0197) | 0.739 | 0.887 |
| rs72499174 | C/G | -0.0054 (0.00589) | 0.36 | 0.67 | 0.0213 (0.0126) | 0.0902 | 0.541 | 0.0105 (0.0131) | 0.421 | 0.67 | -0.00855 (0.0112) | 0.447 | 0.67 | 0.00444 (0.0234) | 0.849 | 0.849 | 0.00591 (0.023) | 0.797 | 0.849 |
