# Supplementary material for: Investigating the role of the relaxin-3/RXFP3 system in neuropsychiatric disorders and metabolic phenotypes: A candidate gene approach
Source: PLoS One. 2023 Nov 15;18(11):e0294045. doi: 10.1371/journal.pone.0294045 (PMC10651050; doi:10.1371/journal.pone.0294045)
Supplement: S5 Table — Regression models were adjusted for age, age2, sex, genotyping batch, testing centre, and the first six European ancestry principal components. Unadjusted p values and q-values (calculated by applying false discovery rate correction across phenotype definitions) are presented. (DOCX) [file pone.0294045.s005.docx]

**Supplementary Table 5:** Full associations between each candidate SNP and each of the 3 phenotypic definitions for atypical depression. Regression models were adjusted for age, age^2^, sex, genotyping batch, testing centre, and the first six European ancestry principal components. Unadjusted p values and q-values (calculated by applying false discovery rate correction across phenotype definitions) are presented.

| **SNP** | **A1/A2** | **CIDI** | | | **PHQ-9 definition** | | | **PHQ-9 cutoff** | | |
| --- | --- | --- | --- | --- | --- | --- | --- | --- | --- | --- |
|  |  | **B (Std. Error)** | ***P*** | **q-value** | **B (Std. Error)** | ***P*** | **q-value** | **B (Std. Error)** | ***P*** | **q-value** |
| rs1982632 | A/G | 0.0266 (0.0453) | 0.557 | 0.836 | 0.0651 (0.0875) | 0.457 | 0.836 | -0.0157 (0.0782) | 0.841 | 0.841 |
| rs78161395 | T/G | 0.0389 (0.0483) | 0.42 | 0.63 | 0.0817 (0.0924) | 0.376 | 0.63 | -0.0338 (0.084) | 0.687 | 0.687 |
| rs74400983 | T/C | -0.0239 (0.0785) | 0.761 | 0.761 | -0.304 (0.174) | 0.0804 | 0.241 | -0.128 (0.141) | 0.364 | 0.546 |
| rs6511905 | G/C | 0.0164 (0.0417) | 0.695 | 0.831 | 0.0173 (0.081) | 0.831 | 0.831 | -0.0245 (0.0716) | 0.732 | 0.831 |
| rs9292519 | A/G | -0.0295 (0.0365) | 0.418 | 0.82 | 0.0161 (0.0707) | 0.820 | 0.820 | 0.0278 (0.0616) | 0.652 | 0.82 |
| rs171631 | A/C | -0.00155 (0.0754) | 0.984 | 0.984 | -0.153 (0.157) | 0.329 | 0.984 | -0.0248 (0.13) | 0.848 | 0.984 |
| rs42868 | G/C | -0.0298 (0.0493) | 0.546 | 0.546 | -0.154 (0.1) | 0.125 | 0.188 | -0.193 (0.089) | 0.0297 | 0.089 |
| rs7702361 | A/C | 0.0304 (0.0365) | 0.404 | 0.770 | 0.0208 (0.0709) | 0.770 | 0.770 | 0.0381 (0.0622) | 0.540 | 0.77 |
| rs11264422 | T/A | -0.0348 (0.0378) | 0.357 | 0.536 | -0.0216 (0.0734) | 0.769 | 0.769 | 0.0669 (0.0634) | 0.291 | 0.536 |
| rs62351166 | A/C | 0.0525 (0.0466) | 0.260 | 0.756 | -0.0088 (0.0921) | 0.924 | 0.924 | 0.0526 (0.0788) | 0.504 | 0.756 |
| rs7695640 | G/A | 0.0632 (0.0508) | 0.214 | 0.568 | -0.072 (0.103) | 0.486 | 0.568 | 0.0494 (0.0865) | 0.568 | 0.568 |
| rs11100192 | G/A | 0.0344 (0.156) | 0.825 | 0.854 | -0.0592 (0.321) | 0.854 | 0.854 | -0.0764 (0.282) | 0.786 | 0.854 |
| rs72703633 | C/T | -0.185 (0.183) | 0.311 | 0.484 | 0.2 (0.293) | 0.494 | 0.494 | 0.252 (0.254) | 0.323 | 0.484 |
| rs11793069 | G/A | 0.0216 (0.0358) | 0.547 | 0.937 | -0.0223 (0.07) | 0.75 | 0.937 | -0.00479 (0.0608) | 0.937 | 0.937 |
| rs72499174 | C/G | 0.0211 (0.0417) | 0.612 | 0.894 | -0.0975 (0.0839) | 0.245 | 0.736 | -0.0095 (0.0715) | 0.894 | 0.894 |
