# Supplementary material for: Investigating the role of the relaxin-3/RXFP3 system in neuropsychiatric disorders and metabolic phenotypes: A candidate gene approach
Source: PLoS One. 2023 Nov 15;18(11):e0294045. doi: 10.1371/journal.pone.0294045 (PMC10651050; doi:10.1371/journal.pone.0294045)
Supplement: S6 Table — Regression models were adjusted for age, age2, sex, genotyping batch, testing centre, and the first six European ancestry principal components. Unadjusted p values and q-values (calculated by applying false discovery rate correction across phenotype definitions) are presented. (DOCX) [file pone.0294045.s006.docx]

**Supplementary Table 6:** Full associations between each candidate SNP and each of the 3 phenotypic definitions for anxiety disorders. Regression models were adjusted for age, age^2^, sex, genotyping batch, testing centre, and the first six European ancestry principal components. Unadjusted p values and q-values (calculated by applying false discovery rate correction across phenotype definitions) are presented.

| **SNP** | **A1/A2** | **ICD10-coded** | | | **Probable** | | | **GAD-7 cutoff** | | |
| --- | --- | --- | --- | --- | --- | --- | --- | --- | --- | --- |
|  |  | **B (Std. Error)** | ***P*** | **q-value** | **B (Std. Error)** | ***P*** | **q-value** | **B (Std. Error)** | ***P*** | **q-value** |
| rs1982632 | A/G | 0.0110 (0.0148) | 0.455 | 0.455 | 0.0141 (0.0143) | 0.322 | 0.455 | -0.0254 (0.0282) | 0.367 | 0.455 |
| rs78161395 | T/G | -0.000691 (0.0158) | 0.965 | 0.965 | -0.00866 (0.0153) | 0.571 | 0.942 | -0.0146 (0.0300) | 0.628 | 0.942 |
| rs74400983 | T/C | -0.00715 (0.0254) | 0.778 | 0.778 | -0.0193 (0.0245) | 0.432 | 0.686 | -0.036 (0.0485) | 0.457 | 0.686 |
| rs6511905 | G/C | -0.00906 (0.0136) | 0.506 | 0.506 | -0.0136 (0.0131) | 0.300 | 0.450 | -0.0295 (0.0259) | 0.254 | 0.450 |
| rs9292519 | A/G | -0.0137 (0.0118) | 0.245 | 0.736 | -0.00178 (0.0114) | 0.876 | 0.904 | -0.00268 (0.0223) | 0.904 | 0.904 |
| rs171631 | A/C | -0.0248 (0.0248) | 0.356 | 0.534 | 0.0216 (0.0234) | 0.356 | 0.534 | -0.000426 (0.0462) | 0.993 | 0.993 |
| rs42868 | G/C | 0.00378 (0.0158) | 0.811 | 0.811 | -0.0104 (0.0153) | 0.496 | 0.743 | -0.0328 (0.0303) | 0.279 | 0.743 |
| rs7702361 | A/C | 0.00478 (0.0118) | 0.685 | 0.685 | 0.00640 (0.0114) | 0.574 | 0.685 | 0.0241 (0.0223) | 0.280 | 0.685 |
| rs11264422 | T/A | 0.0311 (0.0122) | 0.0106 | 0.016 | -0.0348 (0.0118) | 0.00318 | 0.010 | -0.0101 (0.0231) | 0.661 | 0.661 |
| rs62351166 | A/C | 0.00868 (0.0152) | 0.568 | 0.568 | 0.0253 (0.0147) | 0.0856 | 0.257 | -0.0229 (0.0292) | 0.433 | 0.568 |
| rs7695640 | G/A | 0.00132 (0.0166) | 0.937 | 0.937 | 0.00372 (0.0161) | 0.817 | 0.937 | -0.00431 (0.0317) | 0.892 | 0.937 |
| rs11100192 | G/A | -0.0516 (0.0534) | 0.334 | 0.906 | 0.00593 (0.0500) | 0.906 | 0.906 | 0.0170 (0.0970) | 0.861 | 0.906 |
| rs72703633 | C/T | 0.0783 (0.0526) | 0.137 | 0.376 | 0.0105 (0.0519) | 0.840 | 0.840 | 0.111 (0.0966) | 0.251 | 0.376 |
| rs11793069 | G/A | 0.00324 (0.0117) | 0.781 | 0.781 | -0.0180 (0.0112) | 0.108 | 0.325 | 0.0215 (0.0220) | 0.329 | 0.493 |
| rs72499174 | C/G | 0.00871 (0.0136) | 0.523 | 0.821 | 0.00734 (0.0131) | 0.575 | 0.821 | 0.00581 (0.0257) | 0.821 | 0.821 |
