# Supplementary material for: Investigating the role of the relaxin-3/RXFP3 system in neuropsychiatric disorders and metabolic phenotypes: A candidate gene approach
Source: PLoS One. 2023 Nov 15;18(11):e0294045. doi: 10.1371/journal.pone.0294045 (PMC10651050; doi:10.1371/journal.pone.0294045)
Supplement: S7 Table — Regression models were adjusted for age, age2, sex, genotyping batch, testing centre, and the first six European ancestry principal components. Unadjusted p values and q-values (calculated by applying false discovery rate correction across phenotype definitions) are presented. (DOCX) [file pone.0294045.s007.docx]

| **SNP** | **A1/A2** | **Metabolic syndrome** | | | **Hypertension** | | | **Hypertriglyceridaemia** | | | **Low HDL Cholesterol**  **(Dyslipidaemia)** | | | **Hyperglycaemia** | | |
| --- | --- | --- | --- | --- | --- | --- | --- | --- | --- | --- | --- | --- | --- | --- | --- | --- |
|  |  | **B (Std. Error)** | ***P*** | **q-value** | **B (Std. Error)** | ***P*** | **q-value** | **B (Std. Error)** | ***P*** | **q-value** | **B (Std. Error)** | ***P*** | **q-value** | **B (Std. Error)** | ***P*** | **q-value** |
| rs1982632 | A/G | 0.00711 (0.00722) | 0.325 | 0.568 | 0.00801 (0.00739) | 0.278 | 0.568 | 0.00597 (0.00668) | 0.372 | 0.568 | -0.00599 (0.008) | 0.454 | 0.568 | 0.00571 (0.012) | 0.633 | 0.633 |
| rs78161395 | T/G | -0.00229 (0.00771) | 0.766 | 0.8 | -0.00295 (0.00786) | 0.708 | 0.8 | 0.00204 (0.00712) | 0.775 | 0.8 | 0.00216 (0.00853) | 0.8 | 0.8 | -0.015 (0.0128) | 0.244 | 0.800 |
| rs74400983 | T/C | -0.000772 (0.0123) | 0.95 | 0.995 | -0.000082 (0.0126) | 0.995 | 0.995 | -0.00849 (0.0114) | 0.457 | 0.867 | 0.00876 (0.0136) | 0.52 | 0.867 | -0.0198 (0.0206) | 0.336 | 0.867 |
| rs6511905 | G/C | -0.00326 (0.00662) | 0.623 | 0.779 | -0.00189 (0.00675) | 0.779 | 0.779 | 0.00305 (0.00612) | 0.618 | 0.779 | 0.0048 (0.00732) | 0.512 | 0.779 | -0.0157 (0.011) | 0.154 | 0.768 |
| rs9292519 | A/G | -0.00726 (0.00573) | 0.205 | 0.721 | -0.000269 (0.00585) | 0.963 | 0.963 | 0.00053 (0.0053) | 0.92 | 0.963 | -0.0061 (0.00635) | 0.337 | 0.721 | -0.00747 (0.00952) | 0.433 | 0.721 |
| rs171631 | A/C | -0.00176 (0.0119) | 0.882 | 0.972 | -0.00042 (0.0122) | 0.972 | 0.972 | -0.0105 (0.0111) | 0.343 | 0.857 | -0.0244 (0.0133) | 0.0663 | 0.331 | -0.00142 (0.0198) | 0.943 | 0.972 |
| rs42868 | G/C | -0.00507 (0.00773) | 0.512 | 0.512 | 0.013 (0.0079) | 0.0988 | 0.165 | -0.0144 (0.00715) | 0.0445 | 0.111 | -0.0188 (0.00859) | 0.0289 | 0.111 | -0.00875 (0.0128) | 0.496 | 0.512 |
| rs7702361 | A/C | 0.0078 (0.00575) | 0.175 | 0.437 | -0.00606 (0.00587) | 0.302 | 0.503 | 0.002 (0.00532) | 0.707 | 0.707 | 0.00937 (0.00636) | 0.141 | 0.437 | 0.00759 (0.00953) | 0.425 | 0.532 |
| rs11264422 | T/A | -0.00233 (0.00595) | 0.696 | 0.87 | -0.00598 (0.00608) | 0.325 | 0.542 | 0.00951 (0.00551) | 0.0846 | 0.251 | 0.0108 (0.00659) | 0.101 | 0.251 | -0.00075 (0.00988) | 0.939 | 0.939 |
| rs62351166 | A/C | 0.00453 (0.00742) | 0.542 | 0.542 | 0.0055 (0.00757) | 0.468 | 0.542 | 0.00934 (0.00685) | 0.173 | 0.542 | -0.00754 (0.00821) | 0.358 | 0.542 | -0.0129 (0.0123) | 0.297 | 0.542 |
| rs7695640 | G/A | -0.00726 (0.00812) | 0.371 | 0.371 | -0.0102 (0.00829) | 0.219 | 0.293 | -0.0125 (0.00752) | 0.0953 | 0.293 | -0.0107 (0.00902) | 0.235 | 0.293 | -0.0163 (0.0135) | 0.227 | 0.293 |
| rs11100192 | G/A | 0.00814 (0.0256) | 0.75 | 0.75 | -0.0266 (0.0259) | 0.304 | 0.539 | -0.0255 (0.0237) | 0.281 | 0.539 | 0.0227 (0.0281) | 0.418 | 0.539 | 0.033 (0.0419) | 0.431 | 0.539 |
| rs72703633 | C/T | 0.00986 (0.0264) | 0.709 | 0.824 | 0.0397 (0.027) | 0.141 | 0.706 | -0.0143 (0.0247) | 0.562 | 0.824 | -0.0119 (0.0295) | 0.686 | 0.824 | -0.00983 (0.0441) | 0.824 | 0.824 |
| rs11793069 | G/A | 0.00958 (0.00567) | 0.0913 | 0.228 | 0.0122 (0.0058) | 0.0353 | 0.176 | 0.00686 (0.00525) | 0.192 | 0.319 | 0.00298 (0.00628) | 0.635 | 0.635 | -0.00739 (0.00941) | 0.432 | 0.540 |
| rs72499174 | C/G | -0.00974 (0.00665) | 0.143 | 0.238 | -0.0182 (0.00677) | 0.00709 | 0.0355 | -0.0135 (0.00616) | 0.0282 | 0.0704 | -0.00165 (0.00736) | 0.823 | 0.823 | 0.0052 (0.011) | 0.637 | 0.796 |

**Supplementary Table 7:** Full associations between each candidate SNP and metabolic syndrome, as well as 4 sub-outcomes that comprise metabolic syndrome. Regression models were adjusted for age, age^2^, sex, genotyping batch, testing centre, and the first six European ancestry principal components. Unadjusted p values and q-values (calculated by applying false discovery rate correction across phenotype definitions) are presented.
