# Supplementary material for: Investigating the role of the relaxin-3/RXFP3 system in neuropsychiatric disorders and metabolic phenotypes: A candidate gene approach
Source: PLoS One. 2023 Nov 15;18(11):e0294045. doi: 10.1371/journal.pone.0294045 (PMC10651050; doi:10.1371/journal.pone.0294045)
Supplement: S8 Table — Models were adjusted for age, age2, sex, genotyping batch, testing centre, and the first six European ancestry principal components. Unadjusted p values and q-values (calculated by applying false discovery rate correction across phenotype definitions) are presented. (DOCX) [file pone.0294045.s008.docx]

**Supplementary Table 8:** Results of a multivariate regression model with all candidate SNPs at a particular gene as simultaneous explanatory variables and several depression and atypical depression phenotype outcomes, adjusted for age, age^2^, sex, genotyping batch, testing centre, and the first six European ancestry principal components. Unadjusted p values and q-values (calculated by applying false discovery rate correction across phenotype definitions) are presented.

| **Phenotype** | **Gene** | **Chi-square** | ***P*** | **q-value** |
| --- | --- | --- | --- | --- |
| Broad depression | RLN3 | 10.00 | 0.0404 | 0.242 |
|  | RXFP3 | 5.31 | 0.257 | 0.745 |
|  | RXFP1 | 7.14 | 0.0676 | 0.406 |
|  | RLN2 | 2.64 | 0.451 | 0.934 |
| ICD10-coded depression | RLN3 | 0.62 | 0.961 | 0.961 |
|  | RXFP3 | 1.95 | 0.745 | 0.745 |
|  | RXFP1 | 1.36 | 0.715 | 0.859 |
|  | RLN2 | 4.23 | 0.238 | 0.934 |
| Lifetime depression | RLN3 | 3.26 | 0.516 | 0.927 |
|  | RXFP3 | 3.08 | 0.544 | 0.745 |
|  | RXFP1 | 2.50 | 0.476 | 0.859 |
|  | RLN2 | 2.02 | 0.569 | 0.934 |
| CIDI depression | RLN3 | 2.65 | 0.618 | 0.927 |
|  | RXFP3 | 5.90 | 0.206 | 0.745 |
|  | RXFP1 | 2.97 | 0.397 | 0.859 |
|  | RLN2 | 0.71 | 0.871 | 0.934 |
| PHQ-9 definition depression | RLN3 | 1.64 | 0.801 | 0.961 |
|  | RXFP3 | 2.40 | 0.663 | 0.745 |
|  | RXFP1 | 1.71 | 0.636 | 0.859 |
|  | RLN2 | 0.43 | 0.934 | 0.934 |
| PHQ-9 cutoff depression | RLN3 | 2.99 | 0.559 | 0.927 |
|  | RXFP3 | 4.06 | 0.398 | 0.745 |
|  | RXFP1 | 0.50 | 0.92 | 0.920 |
|  | RLN2 | 0.61 | 0.894 | 0.934 |
| CIDI atypical depression | RLN3 | 1.78 | 0.777 | 0.777 |
|  | RXFP3 | 4.69 | 0.321 | 0.481 |
|  | RXFP1 | 3.10 | 0.377 | 0.871 |
|  | RLN2 | 2.11 | 0.55 | 0.796 |
| PHQ-9 definition atypical depression | RLN3 | 6.38 | 0.173 | 0.516 |
|  | RXFP3 | 2.88 | 0.578 | 0.578 |
|  | RXFP1 | 0.71 | 0.871 | 0.871 |
|  | RLN2 | 2.79 | 0.426 | 0.796 |
| PHQ-9 cutoff atypical depression | RLN3 | 4.49 | 0.344 | 0.516 |
|  | RXFP3 | 5.75 | 0.219 | 0.481 |
|  | RXFP1 | 1.27 | 0.737 | 0.871 |
|  | RLN2 | 1.02 | 0.796 | 0.796 |
