# Supplementary material for: Investigating the role of the relaxin-3/RXFP3 system in neuropsychiatric disorders and metabolic phenotypes: A candidate gene approach
Source: PLoS One. 2023 Nov 15;18(11):e0294045. doi: 10.1371/journal.pone.0294045 (PMC10651050; doi:10.1371/journal.pone.0294045)
Supplement: S9 Table — Models were adjusted for age, age2, sex, genotyping batch, testing centre, and the first six European ancestry principal components. Unadjusted p values and q-values (calculated by applying false discovery rate correction across phenotype definitions) are presented. (DOCX) [file pone.0294045.s009.docx]

**Supplementary Table 9:** Results of a multivariate regression model with all candidate SNPs at a particular gene as simultaneous explanatory variables and several anxiety phenotype outcomes, adjusted for age, age^2^, sex, genotyping batch, testing centre, and the first six European ancestry principal components. Unadjusted p values and q-values (calculated by applying false discovery rate correction across phenotype definitions) are presented.

| **Phenotype** | **Gene** | **Chi-square** | ***P*** | **q-value** |
| --- | --- | --- | --- | --- |
| ICD10-coded anxiety | RLN3 | 2.54 | 0.638 | 0.676 |
|  | RXFP3 | 2.91 | 0.573 | 0.676 |
|  | RXFP1 | 1.53 | 0.676 | 0.676 |
|  | RLN2 | 2.08 | 0.556 | 0.794 |
| Lifetime disorder anxiety | RLN3 | 1.68 | 0.794 | 0.794 |
|  | RXFP3 | 8.18 | 0.0853 | 0.256 |
|  | RXFP1 | 2.97 | 0.396 | 0.566 |
|  | RLN2 | 2.69 | 0.442 | 0.566 |
| GAD-7 cutoff anxiety | RLN3 | 2.95 | 0.566 | 0.566 |
|  | RXFP3 | 2.41 | 0.661 | 0.856 |
|  | RXFP1 | 0.77 | 0.856 | 0.856 |
|  | RLN2 | 2.27 | 0.518 | 0.856 |
