# Supplementary material for: Investigating the role of the relaxin-3/RXFP3 system in neuropsychiatric disorders and metabolic phenotypes: A candidate gene approach
Source: PLoS One. 2023 Nov 15;18(11):e0294045. doi: 10.1371/journal.pone.0294045 (PMC10651050; doi:10.1371/journal.pone.0294045)
Supplement: S10 Table — Models were adjusted for age, age2, sex, genotyping batch, testing centre, and the first six European ancestry principal components. (DOCX) [file pone.0294045.s010.docx]

**Supplementary Table 10:** Results of a multivariate regression model with all candidate SNPs at a particular gene as simultaneous explanatory variables and metabolic syndrome, as well as the sub-outcomes comprising the disorder, adjusted for age, age^2^, sex, genotyping batch, testing centre, and the first six European ancestry principal components.

| **Phenotype** | **Gene** | **Chi-square** | ***P*** | **q-value** |
| --- | --- | --- | --- | --- |
| Metabolic syndrome | RLN3 | 1.34 | 0.854 | 0.894 |
|  | RXFP3 | 2.68 | 0.613 | 0.766 |
|  | RXFP1 | 1.12 | 0.773 | 0.773 |
|  | RLN2 | 3.41 | 0.333 | 0.555 |
| Hypertension | RLN3 | 1.37 | 0.85 | 0.894 |
|  | RXFP3 | 7.60 | 0.107 | 0.521 |
|  | RXFP1 | 2.38 | 0.497 | 0.622 |
|  | RLN2 | 11.20 | 0.0109 | 0.054 |
| Hypertriglyceridaemia | RLN3 | 6.89 | 0.142 | 0.710 |
|  | RXFP3 | 4.76 | 0.313 | 0.521 |
|  | RXFP1 | 5.63 | 0.131 | 0.589 |
|  | RLN2 | 5.62 | 0.132 | 0.330 |
| Low HDL cholesterol  (Dyslipidaemia) | RLN3 | 1.10 | 0.894 | 0.894 |
|  | RXFP3 | 4.86 | 0.302 | 0.521 |
|  | RXFP1 | 3.26 | 0.353 | 0.589 |
|  | RLN2 | 0.53 | 0.912 | 0.912 |
| Hyperglycaemia | RLN3 | 4.30 | 0.367 | 0.894 |
|  | RXFP3 | 1.06 | 0.901 | 0.901 |
|  | RXFP1 | 3.60 | 0.308 | 0.589 |
|  | RLN2 | 0.77 | 0.856 | 0.912 |
